# Relationships between daily emotional experiences and smartphone addiction among college students: moderated mediating role of gender and mental health problems

**DOI:** 10.3389/fpsyg.2024.1490338

**Published:** 2024-12-12

**Authors:** Qiuping Cheng, Ying Zhou, Hongying Zhu, Qunlong Wang, Wei Peng

**Affiliations:** ^1^Insititute of Modern Services, Zhejiang Shuren University, Hangzhou, China; ^2^Mental Health Education and Counseling Center, Jinhua University of Vocational Technology, Jinhua, China; ^3^School of Economics and Social Welfare, Zhejiang Shuren University, Hangzhou, China

**Keywords:** smartphone addiction, daily emotional experiences, anxiety, stress, depression, gender

## Abstract

**Introduction:**

The theoretical model of smartphone addiction highlights the role of emotional factors in fostering addictive behaviors. However, most research has focused on long-term emotional states and pathologies, often overlooking the immediate effects of daily emotional fluctuations on smartphone usage and their mechanisms.

**Methods:**

Our study employed an online survey and a moderated parallel mediation model to explore how daily emotional experiences influence smartphone addiction among college students. We analyzed the mediating roles of anxiety, stress, and depression, and the moderating effect of gender.

**Results:**

Our findings indicate that daily negative emotional experiences were positively correlated with smartphone addiction, with stress serving as a significant mediator in the relationship between both positive and negative emotional experiences and addiction. Interestingly, positive emotional experiences directly increased smartphone addiction risk among female students, but they also significantly reduced stress and depression, especially pronounced in women. Further analysis indicated that positive emotions primarily mitigate addiction through reducing stress, a pathway especially significant in females.

**Discussion:**

The study not only confirms the substantial impact of emotional experiences on addiction but also deepens our understanding of their mechanisms, underlining the importance of considering the nature of emotional experiences and gender-specific effects in devising prevention and intervention strategies.

## Introduction

In today’s digital age, smartphones are essential for communication and daily life (Hargittai and Walejko, 2008), enabling people to manage daily affairs, maintain social connections, and enjoy digital conveniences (Westlund, 2015). Research indicates that while smartphones increase convenience and offer psychological benefits (Best et al., 2014; Kim et al., 2017; Valkenburg et al., 2022; Yang C. et al., 2021), their excessive use can lead to risks like addiction (Aka et al., 2020; Billieux et al., 2015; Elhai et al., 2017).

Smartphone addiction, also termed smartphone dependency or problematic smartphone use, involves uncontrollable, prolonged use that leads to physiological, psychological, and social dysfunction (Billieux et al., 2015; Billieux et al., 2008; Ting and Chen, 2020). The phenomenon of smartphone addiction among college students is particularly concerning. Surveys indicate that the rate of smartphone addiction among Chinese college students increased from 4.05 to 27.4% in 2011 to 22.4 to 33.1% in 2022. About 40% of Chinese college students experience varying degrees of smartphone addiction, making it a major non-substance addiction (Keke et al., 2022). Smartphone addiction impairs learning, living, and employment prospects for college students and can trigger psychological, physiological, and social issues (Chen et al., 2016; De-Sola Gutiérrez et al., 2016; Gao et al., 2020; Zou et al., 2022). Therefore, researching smartphone addiction among college students and its underlying mechanisms is crucial for higher education institutions focusing on talent cultivation.

Existing research has predominantly focused on smartphone addiction and its impact on mental health (Elhai et al., 2017; De-Sola Gutiérrez et al., 2016; Canale et al., 2019; Candussi et al., 2023; Elhai et al., 2018b; Elhai and Contractor, 2018; Elhai et al., 2020; Rozgonjuk and Elhai, 2021; Zsila and Reyes, 2023). For instance, Horvath et al. (2020) found that smartphone addiction is associated with reduced gray matter in brain areas such as the left anterior insula, inferior temporal lobe, and hippocampus, which are closely linked to addictive behaviors. Furthermore, smartphone addiction is closely related to mental health issues such as anxiety, depression, sleep problems, and feelings of loneliness (Ratan et al., 2021; Volungis et al., 2020). Lei et al. (2020) further demonstrated that smartphone addiction significantly increases levels of depression, anxiety, and stress among college students. Researchers have long been exploring the factors influencing smartphone addiction, identifying individual psychological traits, social environmental factors, and technological characteristics as critical components influencing the frequency of smartphone use and addictive behaviors (Billieux et al., 2015; Chiu Shao, 2014; Kim et al., 2016). In recent years, the scope of this research has expanded to include the impact of emotional factors on smartphone addiction, highlighting emotions as significant contributors to addictive behaviors.

Theories such as the Compensatory Internet Use Theory (CIUT) (Kardefelt-Winther, 2014), the Integrated Pathways Model (IPM) (Billieux et al., 2015), and the Interaction of Person-Affect-Cognition-Execution (I-PACE) model (Brand et al., 2016; Brand et al., 2019) unanimously indicate that emotions play a central role in the process of smartphone addiction. In these models, emotions are seen as subjective experiences or internal emotional states associated with specific behaviors. Particularly in the face of intense emotions, some individuals may lose control over their actions, ignoring the potential consequences of excessive smartphone use and even seeking high levels of stimulation. These individuals often rely on smartphones to regulate their emotions (Elhai and Contractor, 2018; Rozgonjuk and Elhai, 2021; Elhai et al., 2016; Elhai et al., 2018a). Although these theoretical models provide a framework for understanding how emotional factors influence smartphone usage, existing research primarily focuses on the effects of long-term emotional states and pathological conditions, overlooking the immediate impact of daily emotional fluctuations on smartphone use behavior.

Studies show that daily emotional experiences significantly influence smartphone use trends (Hyvärinen and Beck, 2018; Jo and Baek, 2023a; Jo and Baek, 2023b), and different emotional experiences may increase addiction risks through different mechanisms (Fu et al., 2020; Jin and Oh, 2021). Especially in the early stages of behavioral addiction, both positive and negative emotions can trigger emotional and cognitive responses, enhancing attention to specific stimuli and thereby prompting impulsive smartphone use (Brand et al., 2019; Starcke et al., 2018). Positive emotions, such as feeling enthusiastic, active, and alert, are often associated with pleasant interactions in the environment, which can expand an individual’s momentary thought-action repertoire, promoting exploratory behavior and open-mindedness (Fredrickson, 2001; Watson et al., 1988). From the perspective of habit formation theory (Lally et al., 2010; Lally and Gardner, 2013), repeated experiences of positive emotions during smartphone use can reinforce this behavior, gradually forming a habit that becomes automatic over time, leading to increased usage frequency and potentially addiction. Wood and Neal (2007) emphasized that habits are responses triggered by environmental cues (Neal et al., 2006), meaning that behaviors persist because they have been rewarded in the past (Verplanken and Orbell, 2003; Ouellette and Wood, 1998). When individuals experience positive emotions (such as joy or satisfaction) after using their phones, the association between the emotional reward and phone use strengthens, making the behavior more automatic. Social validation theory further suggests that individuals are motivated to engage in behaviors that receive positive feedback and social approval (Nadkarni and Hofmann, 2011; Benitez et al., 2022). Likes, comments, and affirmations further reinforce smartphone use as a means of gaining social validation, increasing the risk of over-reliance and addiction (Aka et al., 2020; Jo and Baek, 2023a; Jo and Baek, 2023b; Chen et al., 2015). Conversely, negative emotions such as anger, contempt, disgust, guilt, fear, and tension, which induce fear of missing out, may prompt individuals to use smartphones as a coping mechanism to alleviate anxiety, stress, or other adverse emotional states, thereby increasing addiction risks (Kardefelt-Winther, 2014; Cui et al., 2023; Long et al., 2016). Studies have also found that, regardless of whether the emotional experiences are positive or negative, a high level of fear of missing out is significantly present, further exacerbating the risk of smartphone addiction (Li et al., 2020; Przybylski et al., 2013). Therefore, this study aims to delve into how daily emotional experiences influence excessive smartphone use through specific psychological mechanisms.

Traditional medical models often define “mental health” as a state devoid of negative psychological characteristics such as depression, anxiety, and anger. This definition measures health levels by identifying and eliminating psychological pathologies, viewing mental unhealthiness as a direct manifestation of psychological symptoms that hinder individual functioning and quality of life (Diener et al., 2009; Ryff and Keyes, 1995). The “Chinese National Mental Health Development Report (2021—2022)” indicates that 21.48% of college students are at risk of depression, and 45.28% are at risk of anxiety, with undergraduates exhibiting poorer mental health conditions than their junior college counterparts (Kong et al., 2023). Recent meta-analyses also point out that internalizing problems such as depression and anxiety are more severe than externalizing problems among Chinese college students, with detection rates of 20.8 and 13.7%, respectively, (Chen et al., 2022). This is primarily due to rapid emotional changes during the transition from adolescence to adulthood, incomplete maturation of regulatory abilities, and internal and external pressures making students susceptible to psychological disturbances. Research has found that positive emotional experiences can not only effectively enhance the mental health levels of college students (Junça-Silva et al., 2022), but also significantly buffer the effects of stress and depression (Benson and Ong, 2020). Moreover, there is a close correlation between negative emotional experiences and mental health problems, indicating that the nature of emotional experiences has profound effects on the mental health of college students. Poor mental health conditions, including anxiety, depression, and stress, can lead individuals to frequently use smartphones as a means of escaping reality, engaging in social comparison, or seeking information, thereby increasing the risk of smartphone addiction (Ge et al., 2023; Squires et al., 2021; Thomée et al., 2011). Recent studies show that depression symptoms, potential social anxiety disorders, loneliness, family conflicts, and academic pressures are considered significant risk factors for smartphone addiction (Wang et al., 2022; Wang et al., 2021). Enhancing mental health levels can strengthen individual self-control and emotional regulation abilities, helping to resist the temptation of excessive smartphone use (Arpaci, 2021; Kumcagiz and Gunduz, 2016; Li et al., 2021; Markowitz et al., 2019). Furthermore, a healthy psychological state can promote students’ active participation in social activities in real life (Eime et al., 2013; Wei, 2022), and high social support can mitigate the impact of depression on smartphone addiction (Yang X. et al., 2023; Zhao et al., 2021). Therefore, this study hypothesizes that mental health problems mediate the relationship between daily emotional experiences and smartphone addiction.

Currently, research examining gender as a moderating factor in the formation mechanisms of smartphone addiction is relatively scarce (Chen et al., 2017). Previous studies suggest that gender may play a moderating role in the process by which emotional experiences influence smartphone addiction. For instance, research has found that women display more emotional expressiveness in expressing positive emotions and internalizing negative emotions (such as anger and sadness) than men (Chaplin, 2015), while men benefit more from emotional suppression (Mink et al., 2023). This gender difference in emotional experience may make women more reliant on smartphones to regulate emotions and maintain social relationships (Andreassen et al., 2017; Dhir et al., 2016; Niskier et al., 2024; Wei et al., 2023; Zhao et al., 2022), leading to a generally higher rate of smartphone addiction among women (Jo and Baek, 2023a; Ge et al., 2023; Choi et al., 2015; Rozgonjuk et al., 2023; Yue et al., 2021). Conversely, men are more likely to choose video games or sports activities over social media or smartphones to cope with various psychological issues (Niskier et al., 2024; Wei et al., 2023). Recent studies also indicate that emotional imbalance is significantly positively correlated with smartphone addiction in women but not in men (Arpaci and Kocadag, 2020). Furthermore, the habit formation theory (Lally et al., 2010; Wood and Neal, 2007; Neal et al., 2006) and social validation theory (Nadkarni and Hofmann, 2011; Ballara, 2023) also provide insight into gender differences in smartphone addiction. Women, who are generally more emotionally expressive, may find greater reinforcement in the positive emotional experiences associated with smartphone use, such as feelings of social approval and connection (Chaplin, 2015; Li and Zhuo, 2023). This reinforcement makes it easier for women to form habitual smartphone use patterns that become automatic over time. Conversely, men, who are less likely to express emotions openly (Paül et al., 2022), may not experience the same level of emotional reinforcement, leading to less habitual smartphone use. Similarly, social validation theory suggests that women are more motivated by social feedback and validation, which could explain their higher susceptibility to smartphone addiction as they seek affirmation through likes and comments. These gender-based differences in how positive emotions contribute to habit formation and social validation further reinforce the notion that gender plays a moderating role in smartphone addiction.

Moreover, gender may also moderate the impact of daily emotional experiences on mental health. Studies find that women react more intensely to negative emotional stimuli, which is associated with increased risks of depression and anxiety (Stevens and Hamann, 2012). Although the rate of depression among male college students has risen in recent years (Gao et al., 2020), women remain more active and sensitive in emotional expression (Belk and Snell, 1986; Collignon et al., 2010; Plant et al., 2000), suggesting they may benefit more from positive emotional experiences, especially in terms of social interaction and emotional management (Donges et al., 2012; Folkman, 2008). These gender differences may play a key moderating role in the interaction between emotional experiences and mental health problems, thereby influencing the formation of smartphone addiction. Thus, this study hypothesizes that the mediating effects of mental health problems exhibit gender differences.

In summary, this research intends to investigate the moderated mediation effects of mental health problems and gender between daily emotional experiences and smartphone addiction. Specifically, this study aims to examine the mediating role of anxiety, stress, and depression between daily positive/negative emotional experiences and smartphone addiction, and whether these mediations are moderated by gender.

## Methods

### Participants

The present study employed convenience sampling to conduct a questionnaire survey across two universities in Zhejiang, China. The measures and informed consent procedures were completed via the “Questionnaire Star” platform in accordance with the approval of the Ethical Review Committee of Zhejiang Shuren University, ensuring compliance with the ethical standards laid out in the Declaration of Helsinki. After excluding incomplete and invalid responses, a total of 633 valid questionnaires were collected for final analysis. The sample comprised 342 male participants (54%), with an average age of 21.30 years (SD = 0.28), and 291 female participants (46%), with an average age of 22.30 years (SD = 0.27). Regarding academic level distribution, there were 248 first-year students (39.2%), 206 s-year students (32.5%), 155 third-year students (24.5%), and 24 seniors and others (3.8%). To determine the required sample size for the study, G-Power analysis (Faul et al., 2007) was employed, assuming an effect size of 0.02, an *α* level of 0.05, and a desired statistical power of 0.8. The analysis indicated that a minimum of 602 observations was needed.

## Measures

### Daily emotional experiences

The Positive and Negative Affect Schedule (PANAS) (Watson et al., 1988; Crawford and Henry, 2004) was used to measure the frequency of positive and negative emotional experiences among students on a daily basis for 1 week. Each day, participants rated their frequency of emotional experiences using a 5-point Likert scale ranging from 0 (not at all) to 4 (most of the time). The scale includes 10 positive emotions (e.g., amusement, awe, contentment, gratitude, hope, inspiration, interest, joy, love, pride) and nine negative emotions (e.g., anger, shame, fear, disgust, embarrassment, guilt, sadness, contempt, stress). At the end of the week, the daily scores for each emotion were averaged to characterize emotional fluctuations over the period. These averaged scores for each emotion represent the observed variables, which are used to estimate the underlying latent constructs of daily positive emotional experiences and daily negative emotional experiences, respectively. Higher scores on the positive emotions subscale reflect more frequent and/or intense experiences of emotions like joy, gratitude, and hope, while higher scores on the negative emotions subscale indicate more frequent and/or intense experiences of emotions such as anger, fear, and sadness. This approach allows us to capture the typical emotional experiences of participants over the week, which serves as a proxy for their day-to-day emotional experiences. The internal consistency reliability (Cronbach’s *α* coefficients) of the scale was 0.94 overall, with 0.898 for the positive emotions items and 0.88 for the negative emotions items.

### Mental health problems

The short form of the Depression Anxiety Stress Scale (DASS-21) (Crawford and Henry, 2003; Lee, 2019) was employed to evaluate mental health problems. The DASS-21 features 21 items, organized into three subscales: anxiety (DASS-A), stress (DASS-S) and depression (DASS-D), each comprising seven items. The Composite Mental Health Index is defined as a latent variable represented by all 21 items of the DASS-21, which encompass anxiety, stress, and depression. Each subscale’s items serve as observed variables, respectively used to assess the latent variables—anxiety, stress, and depression. Participants rated the extent to which each statement reflected their experiences over the past week using a scoring options ranging from 0 (not applicable) to 3 (very applicable). Higher scores on the anxiety, stress, and depression subscales indicate greater levels of anxiety, experienced stress, and depressive symptoms, respectively. The internal consistency reliability (Cronbach’s α coefficients) were 0.95 for the overall scale, 0.844 for the anxiety subscale, 0.885 for the stress subscale, and 0.883 for the depression subscale.

### Smartphone addiction scale—short version (SAS-SV)

Smartphone addiction was evaluated using the Smartphone Addiction Scale-Short Version (SAS-SV) (Kwon et al., 2013), which consists of 10 items. Each item was rated on a 6-point scale ranging from 1 (“strongly disagree”) to 6 (“strongly agree”). These 10 items serve as observed variables used to represent the latent variable of smartphone addiction. Higher scores on the SAS-SV suggest a greater risk of smartphone addiction. In our study, the internal consistency reliability (Cronbach’s α coefficient) of this scale was 0.89.

### Statistical analysis

Data analysis was conducted using SPSS software version 27.0 and Mplus 8.3 software. Pearson’s product–moment correlation was used to examine the relationships between variables, and structural equation modeling was employed to analyze mediating and moderating effects.

## Results

### Common method biases test

The current study exclusively employed self-report measures to collect data, which might lead to the issue of Common Method Biases. To address this concern, several controls were implemented during the measurement process. These included ensuring the anonymity of respondents, clearly informing participants that the data would be used solely for scientific research purposes, and employing reverse-worded items in some questions to reduce response biases. To further enhance the rigor of the research, a Harman’s single-factor test was conducted prior to data analysis for statistical control, which involves an unrotated principal component factor analysis of all items of the variables. The results showed that there were 8 factors with characteristic roots more significant than 1, and the variance explained by the first factor was 29.505%, which did not exceed the critical standard of 40% (Wen and Ye, 2014). Therefore, it can be considered that there is no common method bias in the variables involved in this study.

### Descriptive analysis and correlation analysis of each variable

Table 1 presents the bivariate correlations, descriptive statistics, and Cronbach’s alphas for 633 college students. Correlational analysis revealed that daily positive emotional experiences correlated with lower levels of negative emotional experiences, anxiety, stress, and depression. Conversely, daily negative emotional experiences correlated with higher levels of anxiety, stress, depression, and increased smartphone addiction. The study also identified significant gender differences in the experiences of daily negative emotions, anxiety, stress, and smartphone addiction among students. These patterns lay the groundwork for further testing of mediating effects.

**Table 1:** Descriptive analysis and correlation analysis of each variable (*n* = 633).

Variable name	M (m ± s.d.)	F (m ± s.d.)	*t*	1	2	3	4	5	6
1. Daily positive emotional experiences	3.44 ± 0.85	3.41 ± 0.81	0.53	(0.898)					
2. Daily negative emotional experiences	2.3 ± 0.74	2.5 ± 0.72	−3.38^**^	−0.12^**^	(0.88)				
3. Anxiety	10.96 ± 3.56	11.69 ± 3.43	−2.63^**^	−0.24^***^	0.56^***^	(0.84)			
4. Stress	11.96 ± 4.08	12.98 ± 4.26	−3.06^**^	−0.29^***^	0.58^***^	0.82^**^	(0.883)		
5. Depression	11.23 ± 4.14	11.55 ± 3.71	−1.02	−0.34^***^	0.54^***^	0.81^**^	0.81^**^	(0.885)	
6. Smartphone addiction	32.02 ± 10.09	35.13 ± 8.77	−4.11^**^	−0.05	0.3^***^	0.46^**^	0.48^**^	0.42^***^	(0.91)

M, male; F, female; m, mean; s.d., standard deviation. Numbers in parentheses are Cronbach’s alphas. ^***^P < 0.001, ^**^P < 0.01, ^*^P < 0.05.

### Testing of mediating effects

This study used structural equation modeling (SEM) to examine the impact of daily emotional experiences on smartphone addiction, accounting for measurement errors and controlling for demographic variables such as gender, age, and grade. The SEM analysis showed an excellent model fit, indicated by *χ*^2^/df = 2.3, AIC = 55773.94, BIC = 56352.5, CFI = 0.93, TLI = 0.92, RMSEA = 0.047, and SRMR = 0.047. The results indicated that daily positive emotional experiences did not significantly predict smartphone addiction (*β* = −0.055, *p* > 0.05). However, daily negative emotional experiences significantly predicted smartphone addiction (*β* = 0.344, *t* = 8.529, SE = 0.04, *p* < 0.001), highlighting them as key drivers of addiction behaviors among college students.

**Figure 1:**
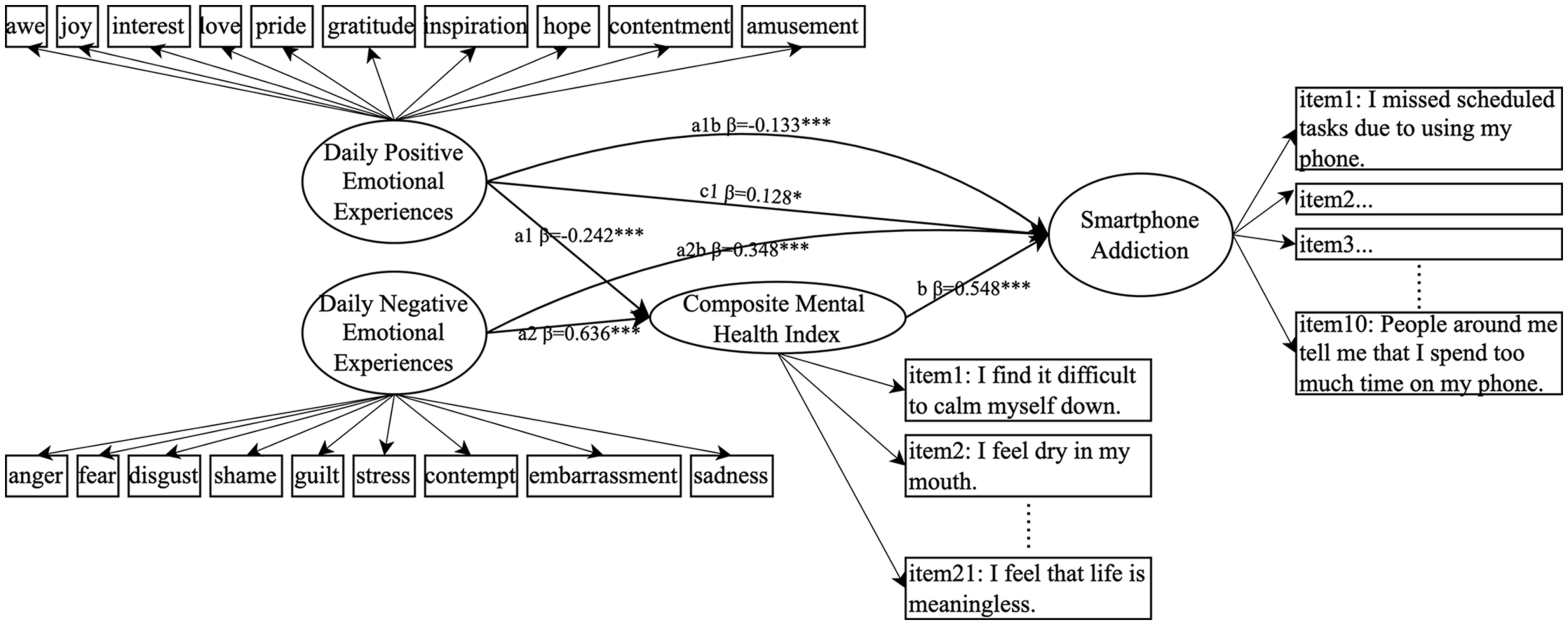
The mediating effect of mental health problems (composite index) between daily emotional experiences and smartphone addiction (*N* = 633). Circles represent latent variables, rectangular boxes represent observed variables, and the data indicate significant standardized path coefficients. *p < 0.05, **p < 0.01, *p < 0.001.

After incorporating the Composite Mental Health Index as a mediator into the model, the resulting path model, as shown in Figure 1, demonstrated a good fit, with *χ*^2^/df = 2.62, CFI = 0.914, TLI = 0.906, RMSEA = 0.051, and SRMR = 0.052. Non-parametric percentile bootstrap analysis with 2000 samples confirmed the significance of the mediation effects. The analysis revealed that daily positive emotional experiences significantly reduced the combined mental health problems (*β* = −0.242, 95% CI = [−0.316, −0.162]), whereas daily negative emotional experiences significantly exacerbated them (*β* = 0.636, 95% CI = [0.563, 0.694]). Furthermore, the Composite Mental Health Index significantly predicted smartphone addiction (*β* = 0.548, 95% CI = [0.413, 0.668]), affirming its robust mediating role. Mediation effect sizes were − 0.133 (95% CI = [−0.187, −0.087]) for positive emotional experiences and 0.348 (95% CI = [0.247, 0.435]) for negative emotional experiences.

The inclusion of mental health as a mediator in the structural equation model showed some notable shifts in the direct effects of both positive and negative emotional experiences on smartphone addiction. Specifically, the direct effect of daily positive emotional experiences on smartphone addiction became statistically significant (*β* = 0.128, 95% CI = [0.03, 0.227]), while the direct effect of negative emotional experiences became insignificant (*β* = −0.003, *p* > 0.05). This change in significance suggests that the influence of positive experiences on smartphone addiction was previously masked by mental health problems, and once these issues were controlled for, the direct impact of positive emotional experiences became clearer, indicating that positive emotions may operate through both direct and indirect pathways. Meanwhile, the insignificance of the direct effect of negative emotions suggested full mediation by mental health problems.

**Figure 2:**
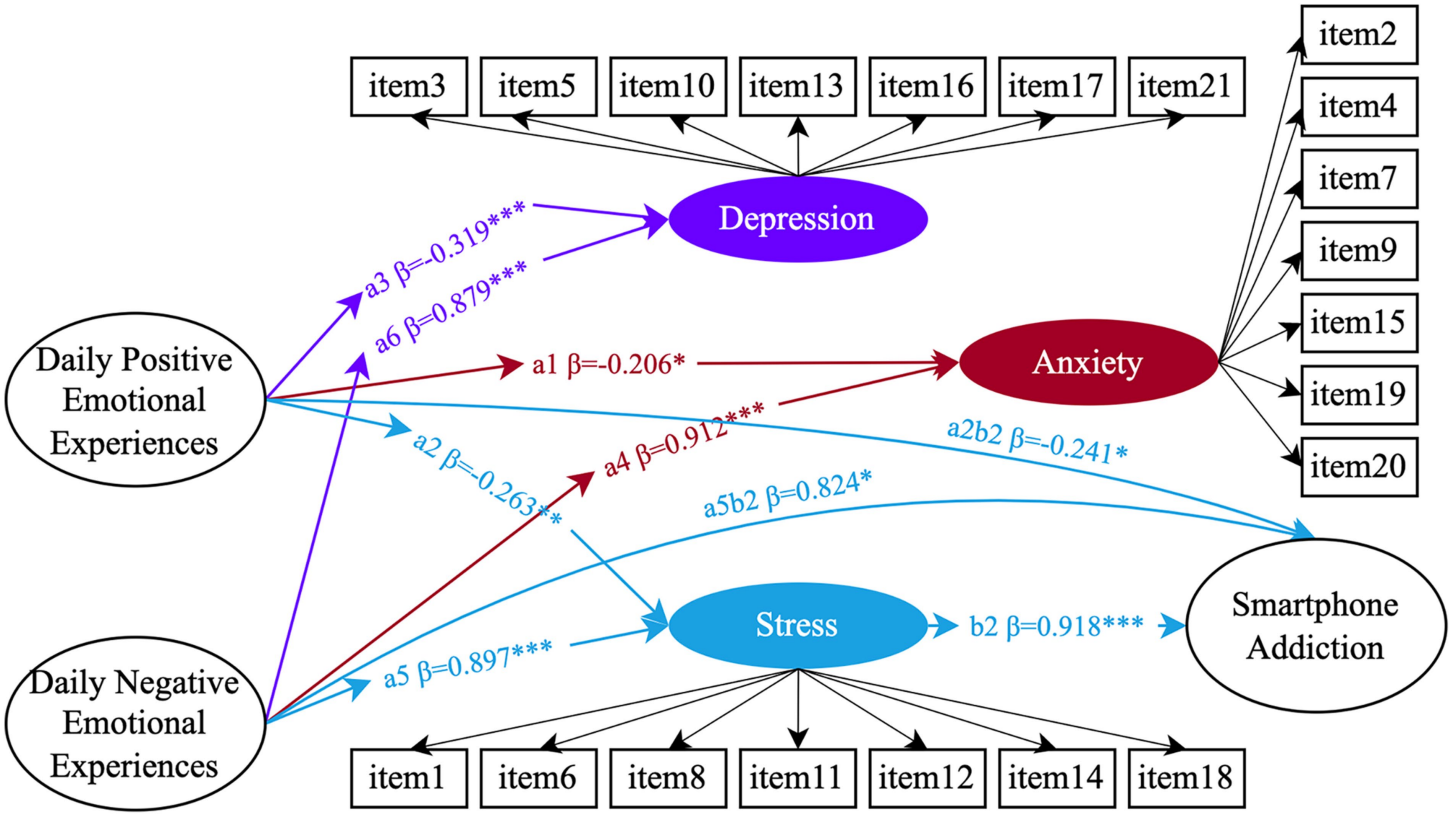
The mediating effects of anxiety (Red), stress (Blue), and depression (purple) between daily emotional experiences and smartphone addiction (*N* = 633). Circles represent latent variables, rectangular boxes represent observed variables, and the data indicate significant standardized path coefficients (*p < 0.05, **p < 0.01, ***p < 0.001). For simplicity and to maintain clarity, the observed variables for daily emotional experiences and smartphone addiction are not displayed in the figure, but are represented similarly to those in Figure 1. In this figure, only the observed variables for the latent constructs of anxiety, stress, and depression are shown, corresponding to the items from the DASS-21 scale.

However, further analysis that examined the components of mental health problems separately—namely anxiety, stress, and depression—revealed a slight decrease in model fit: *χ*^2^/df = 2.937, CFI = 0.9, TLI = 0.89, RMSEA = 0.06, SRMR = 0.06. The resulting path model is shown in Figure 2. Daily positive emotional experiences significantly reduced anxiety (*β* = −0.206, 95% CI = [−0.362, −0.041]), stress (*β* = −0.263, 95% CI = [−0.403, −0.109]), and depression (*β* = −0.319, 95% CI = [−0.467, −0.158]). Conversely, daily negative emotional experiences significantly increased levels of anxiety (*β* = 0.912, 95% CI = [0.83, 0.955]), stress (*β* = 0.897, 95% CI = [0.819, 0.941]), and depression (*β* = 0.879, 95% CI = [0.789, 0.93]). Notably, stress was the only variable that significantly positively predicted smartphone addiction (*β* = 0.918, *p* < 0.001, 95% CI = [0.37, 2.19]). The mediation effects of stress were quantified as −0.241 (95% CI = [−0.703, −0.088]) between positive emotional experiences and smartphone addiction, and 0.824 (95% CI = [0.313, 1.985]) between negative emotional experiences and smartphone addiction.

Compared to the model using the composite mental health index, where positive emotional experiences had a significant direct effect on smartphone addiction, the model with separate components showed different results. When examining anxiety, stress, and depression separately, only stress exhibited a significant mediating effect: negative emotional experiences predominantly escalate smartphone addiction through an intensification of stress, whereas positive emotional experiences substantially mitigate it by reducing stress levels. The direct effects of both positive and negative emotional experiences on smartphone addiction became non-significant in this model, suggesting that stress plays a particularly pivotal role. The combined measure of mental health problems may have obscured the individual contributions of anxiety, stress, and depression, making it difficult to discern the specific role of stress.

### Gender differences in mediation analysis

The study revealed significant gender differences in daily negative emotional experiences, anxiety, stress, and smartphone addiction, with female participants reporting higher incidences than males (daily negative emotional experiences: *t* = −3.38, *p* < 0.01; anxiety: *t* = −2.63, *p* < 0.01; stress: *t* = −3.06, *p* < 0.01; smartphone addiction: *t* = −4.11, *p* < 0.01). However, there were no gender differences in depression, aligning with previous research that indicates traditional symptom-based assessments may not capture gender disparities in depression prevalence (Martin et al., 2013; Nejtek, 2014).

Given that stress mediates the relationship between emotional experiences and smartphone addiction, understanding the role of gender in these mediation pathways is crucial. The varied impacts of emotional experiences and stress responses across genders justify examining gender as a moderating variable, despite the insignificant mediation effects of anxiety and depression. Research indicates that men and women process stress differently, potentially influencing the manifestation of anxiety and depression across genders (Kuehner, 2017; Salk et al., 2017). Moreover, even if mediation is generally not significant, it may become significant when analyzed separately by gender. This analysis might reveal patterns hidden in combined data analyses. For instance, previous studies (Tolin and Foa, 2006) observed that women are more susceptible to stress-related disorders, potentially influencing the mediation effect of stress between emotional experiences and smartphone addiction compared to men. Investigating gender differences can provide deeper insights into how emotional experiences influence smartphone addiction via anxiety, stress, and depression. This approach aligns with calls for nuanced research assessing impacts across different population subgroups (Hyde, 2014), enhancing understanding and effectively tailoring intervention strategies.

### Multi-group analysis

Multi-group analysis was conducted to determine if gender moderates the mediation models involving anxiety, stress, and depression. First, mediation models were analyzed separately for each gender, with both models showing a good fit. The male model (*χ*^2^/df = 1.93, CFI = 0.91, TLI = 0.9, RMSEA = 0.052 [95% CI: 0.049, 0.055], SRMR = 0.058) and the female model (*χ*^2^/df = 1.74, CFI = 0.9, TLI = 0.9, RMSEA = 0.051 [95% CI: 0.047, 0.054], SRMR = 0.056) both met acceptable thresholds, allowing for valid comparisons. Subsequent multi-group comparisons confirmed equivalence models with strong fit indices. Differences in ΔCFI and ΔTLI across models were below 0.01, confirming model validity (see Table 2 for details). This consistency across genders in the mediation model suggests stable significance and latent structures. Although consistent across genders, the model does not rule out specific gender differences in individual pathways or mediations, which further analysis may uncover. Comprehending these differences is essential for understanding gender’s impact on psychological and behavioral patterns.

**Table 2:** Test of measurement invariance for the mediation model across genders (male vs. female).

Model	*χ* ^2^ */df*	CFI	TLI	RMSEA (95% CI)	SRMR	Model compared	ΔCFI	ΔTLI
Gender								
M1 (Configural)	1.78	0.909	0.9	0.05 (0.047, 0.05)	0.058			
M2 (Metric)	1.77	0.908	0.9	0.049 (0.047, 0.05)	0.06	M2 vs. M1	−0.001	0.00
M3 (Scalar)	1.79	0.904	0.9	0.05 (0.048, 0.05)	0.06	M3 vs. M2	−0.004	0.00

### Mediation analysis findings

Mediation analysis revealed that daily positive emotional experiences significantly reduced anxiety, stress, and depression for both men and women. However, the reductions were more moderate for men compared to women. Specifically, men experienced decreases in anxiety (*β* = −0.176, 95% CI = [−0.312, −0.034]), stress (*β* = −0.174, 95% CI = [−0.307, −0.042]), and depression (*β* = −0.269, 95% CI = [−0.408, −0.129]). Women experienced more substantial reductions in anxiety (*β* = −0.337, 95% CI = [−0.494, −0.187]), stress (*β* = −0.449, 95% CI = [−0.588, −0.319]), and depression (*β* = −0.461, 95% CI = [−0.587, −0.335]), suggesting a strong benefit of positive emotional experiences for women. Conversely, daily negative emotional experiences substantially increased anxiety, stress, and depression for both genders. In men, increases were observed in anxiety (*β* = 0.763, 95% CI = [0.611, 0.94]), stress (*β* = 0.791, 95% CI = [0.658, 0.945]), and depression (*β* = 0.756, 95% CI = [0.603, 0.935]). Women experienced similar increases in anxiety (*β* = 0.771, 95% CI = [0.652, 0.88]), stress (*β* = 0.719, 95% CI = [0.594, 0.825]), and depression (*β* = 0.735, 95% CI = [0.62, 0.835]).

### Stress as a mediator

**Figure 3:**
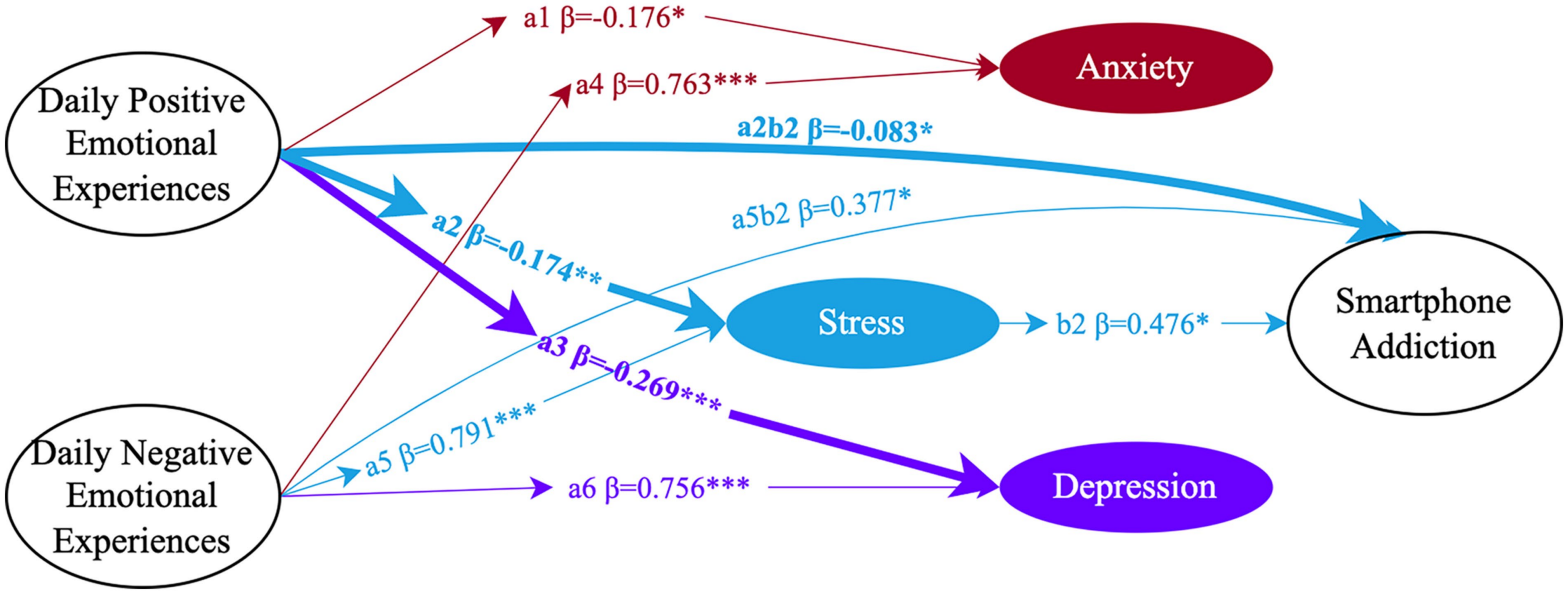
The mediating effects of anxiety (Red), stress (blue), and depression (purple) in male students (*N* = 342). Circles represent latent variables and the data indicate significant standardized path coefficients (*p < 0.05, **p < 0.01, ***p < 0.001). Only latent variables are shown in figure; the corresponding observed variables are omitted for simplicity, which are consistent with Figures 1, 2. Bold solid lines denote significant gender differences in paths.

**Figure 4:**
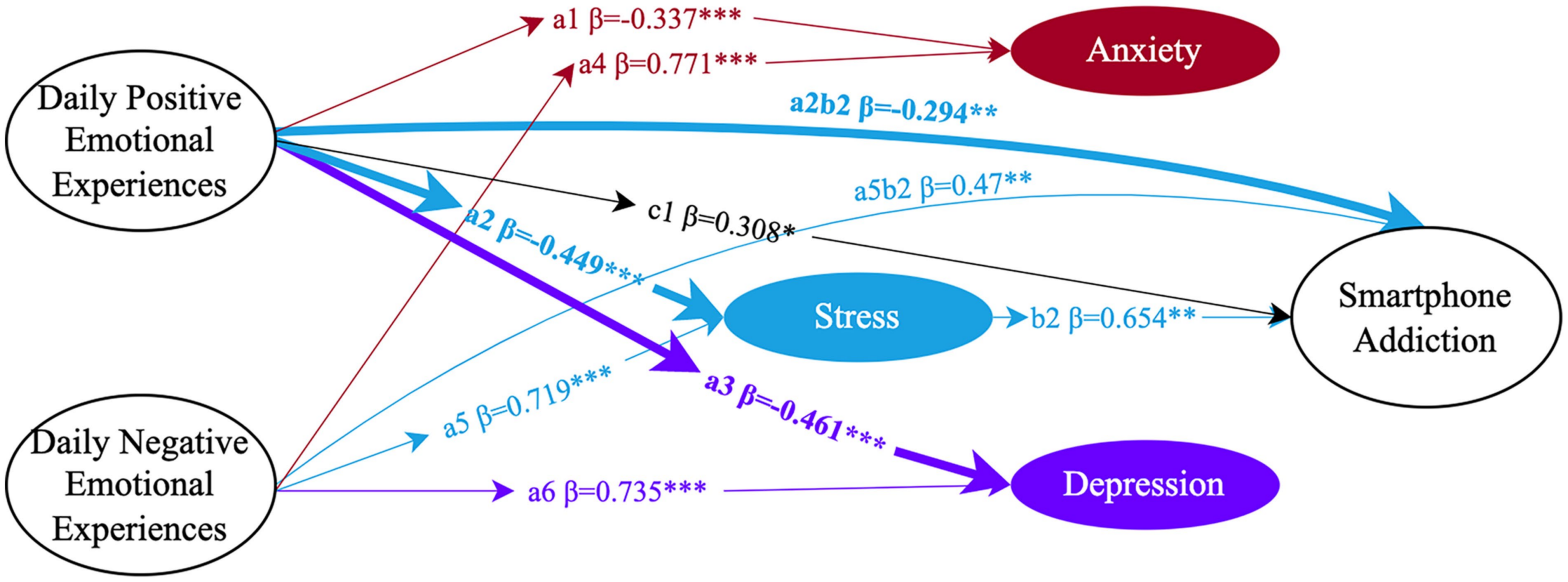
The mediating effects of anxiety (red), stress (Blue), and depression (purple) in female students (*N* = 291). Circles represent latent variables and the data indicate significant standardized path coefficients (*p < 0.05, **p < 0.01, ***p < 0.001). For simplicity, only latent variables are shown in the figure, and the corresponding observed variables are omitted. These observed variables are consistent with those presented in Figures 1, 2. Bold solid lines denote significant gender differences in paths. The black solid line represents the direct effect of daily positive emotional experiences on smartphone addiction, with the black numbers indicating significant direct effects.

Mediation analysis identified stress as the only psychological factor consistently linked to smartphone addiction for both genders. In both men and women, stress was positively correlated with smartphone addiction—men (*β* = 0.476, 95% CI = [0.186, 0.934]) and women (*β* = 0.654, 95% CI = [0.232, 1.099]). The mediation effects of stress varied by the nature of emotional experiences. For men (see Figure 3), stress reduced the likelihood of addiction in the context of positive emotional experiences (*β* = −0.083, 95% CI = [−0.239, −0.017]), while increasing addiction risk during negative emotional experiences (*β* = 0.377, 95% CI = [0.157, 0.985]). For women (see Figure 4), the effects were more pronounced. Stress significantly decreased smartphone addiction with positive emotional experiences (*β* = −0.294, 95% CI = [−0.604, −0.12]) and increased addiction with negative emotional experiences (*β* = 0.47, 95% CI = [0.179, 0.879]). Notably, only in women did daily positive emotional experiences significantly predict smartphone addiction directly (*β* = 0.308, 95% CI = [0.011, 0.851]).

### Critical ratio analysis of gender differences

Analysis of the critical ratio of differences between parameters (CR values) revealed significant gender differences in the effects of daily positive emotional experiences on stress and depression. Additionally, these gender differences extended to the indirect effects of positive emotional experiences through stress on smartphone addiction. The CR values for these pathways were 2.858, 1.981, and 2.614, respectively, all exceeding the threshold of 1.96. This indicates significant gender differences, with gender serving as a moderating factor in these relationships. Specifically, daily positive emotional experiences significantly reduced stress and depression, with more substantial effects observed in females (stress: *β* = −0.449, *p* < 0.001; depression: *β* = −0.461, *p* < 0.001) compared to males (stress: *β* = −0.174, *p* < 0.01; depression: *β* = −0.269, *p* < 0.001). This suggests that females are more responsive to positive emotional influences on stress and depression compared to males.

The indirect path from stress to smartphone addiction further demonstrates the protective effects of positive emotional experiences, with path coefficients of −0.083 for males and − 0.294 for females. This indicates that positive emotional experiences mitigate smartphone addiction more effectively in females by reducing stress.

## Discussion

### Differential impacts of daily emotional experiences on smartphone addiction mediated by mental health problems

The study explored the relationship between daily emotional experiences and smartphone addiction, examining the mediating role of mental health problems and the moderating role of gender. Our findings indicate that daily negative emotional experiences significantly predict smartphone addiction, corroborating prior research that suggests negative emotions foster frequent smartphone use for emotional regulation (Billieux et al., 2015; Elhai et al., 2017; Rozgonjuk and Elhai, 2021; Chiu Shao, 2014; Elhai et al., 2016; Elhai et al., 2018a; Cui et al., 2023; Chen et al., 2022; Yue et al., 2021; Elhai et al., 2019; Shi et al., 2023). Conversely, the impact of positive emotional experiences on smartphone addiction is not significant, possibly due to their influence through multiple mediating pathways with opposing effects, which neutralize each other when mediating variables are not considered, or potentially because the direct influence is masked by these mediators.

Further analysis revealed that a comprehensive set of mental health issues, including anxiety, stress, and depression, mediates the relationship between daily emotional experiences (both negative and positive) and smartphone addiction. Specifically, daily negative emotional experiences positively correlate with this comprehensive set of mental health issues, whereas daily positive experiences exert a negative impact, supporting theories of emotion regulation. According to Gross’s emotion regulation theory (Gross, 1998), emotions significantly influence an individual’s mental health status by shaping their response to stressors (Tsujimoto et al., 2024). Positive emotional experiences effectively mitigate psychological stress and enhance mental wellbeing (Fredrickson, 2001; Leger et al., 2020), while negative emotional experiences can intensify mental health challenges (Hui, 2019). Additionally, this comprehensive set of mental health issues is positively correlated with smartphone addiction, aligning with prior research (Billieux et al., 2015; Elhai and Contractor, 2018; Kardefelt-Winther, 2014; Hyvärinen and Beck, 2018; Cui et al., 2023; Ge et al., 2023; Squires et al., 2021; Thomée et al., 2011) that highlights the role of poor mental health in fostering maladaptive coping mechanisms, such as excessive smartphone use. Poor mental health may lead individuals to frequently use smartphones as a means of escaping reality, engaging in social comparisons, or seeking information, increasing the risk of addiction. Overall, this study underscores mental health issues as pivotal factors in how daily emotional experiences influence smartphone addiction.

In this study, it was observed that daily positive emotional experiences had no significant impact on smartphone addiction when not considering the composite score of anxiety, depression, and stress as a mediating variable. This suggests that positive emotional experiences do not directly contribute to an increase in smartphone addiction. However, when these mental health problems were included as mediators, the influence of positive emotions became significant. This indicates that positive emotions may affect smartphone addiction through non-intuitive mechanisms. Specifically, although positive emotions are generally associated with better mental health, they may also encourage individuals to use smartphones more frequently to maintain or enhance their positive feelings. This finding aligns with previous studies indicating that positive emotions, such as sensation-seeking or perceived enjoyment, can lead to increased smartphone use and potentially raise the risk of addiction (Billieux et al., 2008; Jo and Baek, 2023a; Jo and Baek, 2023b; Chen et al., 2015; Dir and Cyders, 2015; Marengo et al., 2022). These results are consistent with both habit formation and social validation theories. According to habit formation theory (Lally et al., 2010; Lally and Gardner, 2013), repeated positive emotional experiences can make behaviors more automatic, reinforcing habitual smartphone use. Social validation theory further suggests that positive social feedback, such as likes and comments, encourages individuals to engage in smartphone use (Nadkarni and Hofmann, 2011; Benitez et al., 2022).

Conversely, negative emotional experiences were found to significantly influence smartphone addiction, which supports previous findings that negative emotions prompt smartphones use for emotional regulation. However, when mental health problems were included as mediators, the direct effect of negative emotions on smartphone addiction became non-significant. This implies that the impact of negative emotions on smartphone addiction primarily operates indirectly through their effects on mental health. Specifically, negative emotions may first trigger or worsen mental health problems, which then lead to increased smartphone dependency as a coping mechanism or a means of escaping reality (Kardefelt-Winther, 2014; Brand et al., 2016; Brand et al., 2019; Elhai et al., 2019).

### The mediating role of stress between daily emotional experiences and smartphone addiction

Mediation analysis results showed that among the three components of mental health issues—stress, anxiety, and depression—only stress was found to mediate the relationship between daily emotional experiences and smartphone addiction, consistent with previous studies (Wang et al., 2022; Dharmadhikari et al., 2019). Negative emotional experiences escalate stress, increasing addiction risk, while positive experiences alleviate stress, reducing it. Stress, therefore, acts both as an accelerator for negative emotional experiences and as a buffer for positive experiences. These findings are consistent with those of Kassel et al. (2003), who linked negative emotions with psychological stress, and with Billieux et al. (2015), which suggested that using smartphones for emotional regulation can lead to addiction (Billieux et al., 2015). Interpersonal, academic, and employment stresses from negative life events are major sources of stress among college students. These stresses are frequently associated with lower levels of psychological health and more severe smartphone addiction (Wang et al., 2021; Han and Ghazali, 2024; Yang H. et al., 2021; Yang Y. et al., 2023). These findings align with the stress-coping model, which proposes that individuals engage in maladaptive behaviors, such as excessive smartphone use, as a coping mechanism to alleviate discomfort caused by stress.

On the other hand, according to Fredrickson (2001) broaden-and-build theory, positive emotions can expand cognitive and behavioral patterns, promoting the development of enduring resources such as psychological resilience, which reduces the risk of stress and addictive behaviors (Fredrickson, 2001). Studies have shown that interventions enhancing positive emotions are effective in reducing stress among college students (Tsujimoto et al., 2024), boosting their resilience, and subsequently mitigating addictive behaviors (Garland, 2021). These results also align with Wang et al. (2015), who identified perceived stress as a moderator in the relationship between motivations for entertainment, escapism, and smartphone addiction. This suggests that interventions that effectively manage stress might modulate these motivations, reducing addictive behaviors.

Although anxiety and depression significantly affect mental health, we did not find direct effects of these factors on smartphone addiction or significant mediating effects between daily emotional experiences and smartphone addiction. In the I-PACE model, stress is categorized differently from anxiety and depression. Stress refers to subjective situational perception, while anxiety and depression are considered pathological components of core personality traits. This distinction highlights the crucial role of stress in smartphone addiction, while the lack of a mediating role for anxiety and depression may be due to different coping mechanisms. Literature suggests that anxiety and depression often lead to behavioral disengagement and reduced motivation (Burkhardt et al., 2021; Koppe and Rothermund, 2017), whereas stress triggers an immediate need for distraction or relief, which can be fulfilled by smartphone use (van Deursen et al., 2015).

Moreover, the direct influence of stress on smartphone addiction may obscure more intricate and less immediate pathways involving anxiety and depression (Zhao et al., 2022). Research has shown varied and complex associations between anxiety, depression, and smartphone addiction. While some studies identify anxiety as a primary risk factor for smartphone addiction (Dalbudak et al., 2014; Demirci et al., 2015; Keles et al., 2020), with individuals often seeking attention, support, or a sense of belonging through social media to alleviate negative emotions (Vannucci et al., 2017), others did not find this significant relationship (Cui et al., 2023; Yue et al., 2021; Javaeed et al., 2019; Mamun et al., 2019). Similarly, while depressive symptoms are often observed among high-risk smartphone users (Kim et al., 2015), and linked to internet addiction (Zhao et al., 2023), other research shows a weaker relationship (Zhao et al., 2022; Marttila et al., 2021), or even inverse relationship, suggesting depression could act as a protective factor against addiction (Choi et al., 2015). While some studies suggest that smartphone addiction may lead to anxiety and depression, consensus is lacking on whether anxiety and depression also lead to smartphone addiction. This discrepancy highlights the need to focus more on stress regulation rather than solely on anxiety and depression symptoms when designing in interventions for smartphone addiction among college students (Güldal et al., 2022).

### Gender differences in emotional experiences and smartphone addiction

In this study, we found significant gender differences in daily negative emotional experiences, anxiety, stress, and smartphone addiction, with female college students generally exhibiting higher rates than their male counterparts. These findings align with previous research indicating that although women may express emotions more actively, they exhibit stronger tendencies to internalize negative emotions (Chaplin, 2015; Mink et al., 2023; Stevens and Hamann, 2012; van Deursen et al., 2015). We observed no significant gender differences in depressive symptoms, consistent with previous studies suggesting that gender differences in traditional symptoms of depression may not be apparent (Martin et al., 2013; Nejtek, 2014). This result aligns with recent studies indicating a sharp rise in depression rates among male college students (Gao et al., 2020), suggesting that gender differences in depression symptoms may be becoming less pronounced. Consistent with past research, our study also found that rates of smartphone addiction are generally higher among females than males (Jo and Baek, 2023a; Ge et al., 2023; Choi et al., 2015; Rozgonjuk et al., 2023; Yue et al., 2021), possibly linked to women’s more active use of social networks and greater reliance on smartphones for emotional regulation and social interaction (Dhir et al., 2016; Zhao et al., 2022), while men may prefer online gaming (Rozgonjuk et al., 2023).

This study also indicates that gender can modulate the mediating effect of stress between daily positive emotional experiences and smartphone addiction, where stress and gender play a moderated mediating role. Firstly, gender moderates the direct relationship between daily positive emotional experiences and smartphone addiction. Specifically, daily positive emotional experiences can positively predict smartphone addiction in female college students, but not in male college students. Research shows that women are generally more emotionally expressive than men (Chaplin, 2015), whereas men tend to benefit more from emotional suppression (Mink et al., 2023). This difference may lead women to rely more heavily on smartphones for emotional regulation and maintaining social connections, ultimately resulting in higher rates of smartphone addiction among them. Our findings are consistent with the perspectives of habit formation theory and social validation theory concerning gender differences. According to habit formation theory, women may be more susceptible to habitual smartphone use due to their frequent reliance on their devices for emotional regulation, making these behaviors increasing automatic over time. Furthermore, social validation theory suggests that individuals are motivated by positive social feedback, such as likes and comments, which further encourages smartphone use. Given that women are generally more sensitive to social interactions, they may be particularly affected by such positive reinforcement, increasing their risk of addiction. These processes explain why women are more likely to develop addictive smartphone behaviors, emphasizing the significant role gender plays in this context.

Secondly, gender plays a moderating role in the indirect effects of stress from daily positive emotional experiences. Controlling for the same variables, gender analysis reveals consistency in the model between males and females, though differences exist in specific paths and mediating effects. Specifically, women show a more significant direct effect of positive emotional experiences in reducing stress and depression, benefiting more from daily positive emotional experiences. This is consistent with prior research, which shows that women are more active and sensitive in emotional expression (Belk and Snell, 1986; Collignon et al., 2010; Plant et al., 2000), indicating they benefit more from positive emotional experiences, experience less stress, especially in social interactions and emotional management (Donges et al., 2012; Folkman, 2008; Soltani et al., 2017). Furthermore, we found that women also benefit more from daily positive emotional experiences in reducing stress and subsequently lowering the risk of smartphone addiction.

Relatively, both men and women show similar increases in stress from negative emotional experiences and their enhanced impact on smartphone addiction, although previous literature suggests that the types of addiction differ; among women, there is a strong correlation between stress and social media addiction, while among men, gaming and short video addictions are more significantly linked to stress (Andreassen et al., 2017; Niskier et al., 2024; Wei et al., 2023).

### Implications and limitations

This study underscores the pivotal role of daily emotional experiences in smartphone addiction behaviors, particularly how stress acts as a crucial mediator in the relationship between daily emotional fluctuations and smartphone addiction. We observed that negative emotional experiences exacerbate smartphone addiction by increasing stress, while positive emotional experiences counteract addiction by alleviating stress. These findings support the importance of incorporating emotion regulation theories in prevention and intervention strategies, especially for high-risk groups such as females who frequently use smartphones for social interaction and emotional regulation. Moreover, the results highlight how gender influences the relationship between emotional experiences and smartphone addiction, indicating the need to consider gender differences when developing targeted intervention measures.

This study is primarily based on cross-sectional data, which limits our ability to interpret causal relationships. Additionally, the sample consisted only of students from two universities in Zhejiang, which may limit the generalizability of the findings to other populations or cultural contexts. The influence of cultural and regional factors on emotional experiences and smartphone addiction was not extensively explored, which may affect the broader applicability of the results. Future research should employ longitudinal designs to more accurately explore the dynamics between emotional experiences and smartphone addiction, and should also consider expanding the sample to include participants from diverse regions and cultural backgrounds to improve the generalizability and relevance of the findings. Additionally, although we examined the mediating roles of stress, anxiety, and depression in smartphone addiction, we did not find significant mediating effects for anxiety and depression. This may be due to limitations in the measurement tools or sample characteristics used, suggesting that future studies should employ a more diverse approach to assess these mental health issues. Lastly, while this research provides meaningful insights into gender aspects, the study sample’s limited regional scope means that these findings may not be fully applicable to other populations or cultural contexts. Future studies should validate these findings in broader demographics, considering cultural and regional diversity, to enhance the generalizability of the research.

## Conclusion

This study explores the complex relationships between daily emotional experiences, mental health problems, and smartphone addiction, with a focus on gender differences. The findings indicate that negative emotional experiences significantly increase stress, which fully mediates the relationship with smartphone addiction, thereby deepening our understanding of how emotional regulation functions in coping mechanisms for excessive smartphone use. Conversely, positive emotional experiences indirectly reduce smartphone addiction by mitigating stress, highlighting the protective role of positive emotions in preventing addictive behaviors.

The role of stress as both a risk and protective factor highlights the importance of incorporating emotional regulation strategies into intervention programs. Gender differences further enrich these findings, revealing that women are more prone to smartphone addiction compared to men, largely due to their more frequent use of smartphones for social interaction and emotional regulation. Additionally, stress serves as a stronger mediator for women, indicating that positive emotional experiences more effectively reduce stress and curb addictive behaviors in females.

In conclusion, these findings highlight the need to account for emotional triggers and gender-specific mental health conditions in developing preventive and interventional strategies for smartphone addiction among college students. Promoting positive emotional experiences and effectively managing stress can mitigate the risks associated with smartphone addiction and enhance overall mental health. Future research should continue to explore these dynamics, perhaps focusing on longitudinal data to better understand the causality and long-term effects of emotional experiences on smartphone addiction.

## Data Availability

The datasets presented in this study can be found in online repositories. The names of the repository/repositories and accession number(s) can be found at: https://doi.org/10.6084/m9.figshare.26915656.v1.
